# Inhibition of Autophagy Prolongs Recipient Survival Through Promoting CD8^+^ T Cell Apoptosis in a Rat Liver Transplantation Model

**DOI:** 10.3389/fimmu.2019.01356

**Published:** 2019-06-14

**Authors:** Xiaolong Chen, Li Wang, Yinan Deng, Xuejiao Li, Guolin Li, Jing Zhou, Daorou Cheng, Yang Yang, Qing Yang, Guihua Chen, Genshu Wang

**Affiliations:** ^1^Department of Hepatic Surgery, Liver Transplantation, The Third Affiliated Hospital of Sun Yat-sen University, Guangzhou, China; ^2^Guangdong Key Laboratory of Liver Disease Research, The Third Affiliated Hospital of Sun Yat-sen University, Guangzhou, China; ^3^Department of Biliary and Pancreatic Surgery, Sun Yat-sen Memorial Hospital of Sun Yat-sen University, Guangzhou, China; ^4^Department of Pathology, The Third Affiliated Hospital of Sun Yat-sen University, Guangzhou, China

**Keywords:** T cell, liver transplantation, acute rejection, autophagy, apoptosis

## Abstract

In liver transplantation (LT), although various immunosuppressants have been used in clinical practice, acute rejection remains a common complication that significantly shortens recipient survival. In recent years, manipulating immune tolerance has been regarded as one of the promising solutions to rejection. Autophagy, an evolutionarily conserved protein degradation system, has been reported to be involved in immune rejection and may be a target to establish immune tolerance. However, the role of autophagy in acute rejection reaction after LT has not been elucidated. Here, we showed that the autophagy of CD8^+^ T cells was strongly enhanced in patients with graft rejection and that the autophagy level was positively correlated with the severity of rejection. Similar findings were observed in a rat acute hepatic rejection model. Furthermore, administration of the autophagy inhibitor 3-methyladenine (3-MA) largely decreased the viability and function of CD8^+^ T cells through inhibiting autophagy, which significantly prolonged graft survival in rats. In addition, inhibiting the autophagy of activated CD8^+^ T cells *in vitro* considerably suppressed mitochondria mediated survival and downregulated T cell function.

**Conclusions:** We first showed that the inhibition of autophagy significantly prolongs liver allograft survival by promoting the apoptosis of CD8^+^ T cells, which may provide a novel strategy for immune tolerance induction.

## Introduction

Liver transplantation (LT) has become the most effective treatment for patients with end-stage liver disease (ESLD). Although the liver is considered an immunologically privileged organ, the incidence of acute rejection (AR) after LT remains as high as 20–40%, which seriously affects grafts and recipient survival (1). Immunosuppressants are used to alleviate acute rejection to some extent, but notable side effects are observed during lifetime application of modern immunosuppressant. Apart from the significantly increased incidences of renal disease, cardiovascular disease and infections, more than 20% of *de novo* malignancies are reportedly related to immunosuppression (2, 3). Graft immune tolerance refers to the long-term coexistence of the recipient and with the graft in the absence of an immunosuppressant (4). Induction of immune tolerance has been considered the ideal method with less toxicity and more effectiveness. For successful establishment of immunological tolerance, it is necessary to further explore the mechanism of rejection after liver transplantation.

T-cell mediated rejection (TCMR) is a common and important rejection reaction in clinical settings (5). In particular, CD8^+^ T lymphocytes are reportedly the main effector cell subset that plays a critical role in rejection by destroying allograft cells that express the heterogeneous major histocompatibility complex (MHC) (6). After recognizing transplant antigens (mainly foreign MHC I/peptide) and co-stimulatory signals, naïve CD8^+^ T lymphocytes proliferate and differentiate into effector T cells (7), effector CD8^+^ T cells attack transplanted organs by inducing graft parenchymal cell apoptosis through granzyme/perforin release or the Fas-FasL pathway, and by producing inflammatory cytokines that attract neutrophils and/or mononuclear macrophages to induce further damage (8). T cell depletion is thought to be sufficient to induce immune tolerance and has already been realized in some animal models (9–11); however, its translation into in clinical practice remains difficult, and novel therapeutic strategies needed to be established.

Autophagy is an evolutionarily conserved protein degradation system that is essential for cellular homeostasis (12). Under non-stress conditions, autophagy is maintained at relatively low levels to maintain the stability of intracellular metabolism while it can be strongly induced response to starvation or other stresses (13). Autophagy plays a key role in the proliferation, activation and function of T lymphocytes. Autophagy-deficient T lymphocytes are susceptible to apoptosis and exhibit defects in homeostasis and function (14, 15). Some recent studies have also found that autophagy is involved in immune rejection and tolerance after heart transplantation in murine models (16, 17). However, the exact mechanism by which autophagy participates in acute rejection after liver transplantation remains unclear.

In the present study, we found that the autophagy of graft-infiltrated CD8^+^ T cells was strongly enhanced in patients with acute allograft rejection and that the autophagy level of CD8^+^ T cells was positively correlated with rejection severity. We then established an acute rejection model of rat liver transplantation and obtained similar findings. Furthermore, administration of the autophagy inhibitor 3-MA significantly decreased the viability and function of CD8^+^ T cells by inhibiting autophagy, which prolonged graft and recipient survival. In addition, inhibition of the autophagy of activated CD8^+^ T cells *in vitro* largely suppressed mitochondria-mediated survival and IFN-gamma secretion. These results suggest that CD8^+^ T cell autophagy represents a key mechanism underlying acute rejection and may provide a new strategy for immune tolerance induction.

## Materials and Methods

### Clinical Liver Samples

Ten paraffin-embedded liver sections of human liver tissue with different grades of rejection and five control sections with normal liver histology from hepatic hemangioma patients were obtained from the Institute of Pathology at the Third Affiliated Hospital of Sun Yat-sen University. The sections were used for histological and immunohistochemical analysis. Written informed consent was obtained from all the patients in accordance with the ethics committee of the Third Affiliated Hospital of Sun Yat-sen University.

### Animals

MHC mismatched male Lewis (RT11) and male Brown Norway (BN, RT1n) rats were all purchased from Vital River Company (Beijing, China) and housed at the Institute of Laboratory Animal Science, Guangdong Pharmaceutical University. All rats were maintained in a standard environment with a 12/12-h light/dark cycle.

### Orthotopic Liver Transplantation

Lewis rats weighing 210–230 g were used as donors, BN rats weighing 220 g-240 g were used as recipients. An acute rejection model was established by transplanting livers from Lewis rats to BN rats using “two-cuff method” as previously described (18). The transplantation model from BN to BN was used as syngeneic control. The cold ischemia time was (35.0±1.3)min and the warm ischemia time was (0.25±0.05) min, respectively. Some of the recipient rats were sacrificed on day 14 for the collections of liver, peripheral blood and spleen samples. The remaining rats were monitored to assess their survival time. All the experimental procedures were conducted in accordance with the principles and guidelines for the care and use of animals established by Guangdong Pharmaceutical University.

### 3-Methyladenine Administration

The acute hepatic rejection recipient rats were divided into 3-Methyladenine (3-MA, Sigma, St. Louis, MO, USA) treated group and untreated group. The recipient rats in the 3-MA-treated group were intraperitoneally administered 3-MA at a dose of 24 mg/kg every 3 days starting from the day before transplantation. The dosage was reduced by half every 9 days and maintained with a minimum does of 3 mg/kg for 6 days. 3-MA medication was withdrawn 32 days later after LT.

### H&E Staining of Liver Samples

Liver tissues were fixed in 4% paraformaldehyde and embedded in paraffin for 24 h. Four-micron-thick sections were prepared, dewaxed and stained with haematoxylin and eosin (H&E) for histological studies according to standard histological procedures. The acute rejection degree was assessed according to the Banff scheme (19), which includes three individual indexes: (1) damage to bile duct, (2) endothelial inflammation, and (3) portal mononuclear cells infiltration. The rejection activity of all liver sections was assessed by two pathologists independently.

### Immunofluorescent and TUNEL Staining

Immunohistochemistry of liver tissue sections was performed according to the manufacturer's instructions. Briefly, following retrieval in ethylene-diamine -tetraacetic acid (pH 8.0), the liver slides were routinely incubated with primary antibodies against LC3 (Cell Signaling Technology, CA, USA) or CD8 (Sigma-Aldrich, CA, USA) overnight and then stained using an ABC kit (DAKO, USA). For double immunofluorescent staining analysis, liver cryosections were stained with a mixture of rabbit monoclonal anti-LC3 and mouse monoclonal anti-CD8 antibodies and then with a mixture of Alexa Fluor 488-conjugated anti-rabbit IgG and Alexa Fluor 594-conjugated anti-mouse IgG (Molecular Probes, Eugene). TUNEL staining analysis was performed using a Cell Death Kit (KeyGEN BioTECH, Jiangsu, China) according to the manufacturer's instructions. The numbers of immuno-positive cells for LC3, CD8 and TUNEL in 20 random fields under a light microscope (original magnifi-cation: ×200) were counted by a researcher in a blinded manner.

### Enzyme-Linked Immunosorbent Assay

The serum concentrations of inflammatory cytokines (IL-2, IFN-γ, TNF-α) concentrations were determined using a multiplex ELISA assay kit (R&D Systems, Minneapolis, MN, USA) according to previously described procedures (20).

### Western Blotting

Immunoblotting was performed according to the manufacturer's instructions using the following antibodies: anti-LC3, anti-GAPDH and HRP-conjugated goat anti-rabbit IgG antibodies (all the antibodies were obtained from Cell Signaling Technology; Beverly, MA, USA).

### Isolation of Lymphocytes and Flow Cytometry Analysis

Peripheral blood mononuclear cells (PBMCs) were isolated from the peripheral blood of patients and rat recipients by Ficoll density-gradient centrifugation according to the manufacturer's instructions. Liver grafts and spleens from rat recipients were initially cut up carefully, and the liver homogenate need further incubated with a mixture of collagenase type IV (0.5 mg/ml, Sigma-Aldrich, St. Louis, MO, USA) and DNase I (0.1 mg/ml, Roche, Basel, Switzerland) for 1 h at 37°C. The dissociated liver cell suspensions and splenocytes were then filtered through a 75-μm nylon mesh. Mononuclear cells were further isolated using the above-described methods. The surface markers and intracellular proteins of the cells were stained with mAbs against CD4, CD8, Ki67, IFN-gamma, and PI (BD. Pharmingen, San Diego, CA, USA) and analyzed using FACScan and CellQuest software (BD Biosciences Franklin Lakes, NJ, USA).

### Assessment of the Viability, Mitochondrial Stabilization and Function of CD8^+^ T Cells

Lymphocytes isolated from peripheral blood of health donors were plated in 96-well round-bottom plates at 3 ×10^6^ cells per well in RPMI 1640 containing 10% FBS. The cells in some of the wells were activated using anti CD3/28 mAb beads (costimulatory) or a heterologous counterpart (one-way MLR) as previously described (21). In some experiments, the lymphocytes were stained with carboxyfluorescein succinimidyl ester (CFSE) for the assessment of proliferation test as described previously (22). The cells were then treated with 3-MA (5 μM) or the autophagosome degradation inhibitor chloroquine (CQ, 20 μM), incubated for 72 h and harvested for flow cytometry. Cells were harvested for flow cytometry after 72 h of incubation. Cellular apoptosis was determined using an annexin V/propidium iodide (PI) apoptosis detection kit according to the manufacturer's instructions. The apoptosis and proliferation of CD8^+^ T cells were measured using FlowJo software. The mitochondrial mass and mitochondria-associated ROS production was measured according to previously described procedures (23).

### Statistical Analysis

The results are expressed as the mean ± standard deviations (means ± SDs). Student's *t*-test was performed to determine the statistical significance of the differences between groups. The statistical analyses were performed using the statistical software package SPSS 13.0 (SPSS Inc., Chicago IL, USA). A *p* < 0.05 was considered to indicate statistical significance. A Log-rank test was performed to compare the survival times between different groups of recipient rats.

## Results

### Enhanced Autophagy in CD8^+^ T Cells Correlates Positively With the Severity of Acute Rejection in Patients

**Figure 1:**
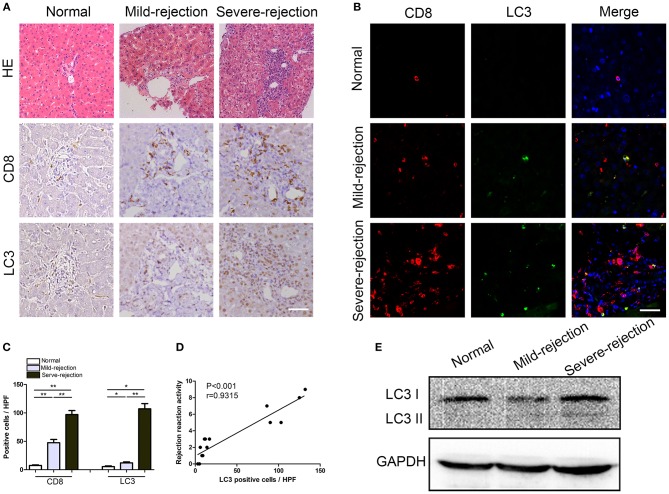
Enhanced autophagy in CD8^+^ T cells correlates positively with the severity of acute rejection in patients.**(A)** Hematoxylin and eosin staining and immunostaining for CD8 and the autophagy marker LC3 in normal and rejection liver samples. Scale bar, 50 μm. **(B)** Double fluorescent staining for CD8 (red), LC3 (green) and DAPI (blue) in liver samples. **(C)** Histogram showing the counts of CD8 positive and LC3 positive cells. **(D)** The correlation between LC3 positive cells and acute rejection severity. **(E)** Expression of LC3 in CD8^+^ T cells isolated from blood was further determined by western blot. All statistical analyses were performed by student *t*-test, *p < 0.05, **p < 0.01 compared between groups.

To investigate the role of autophagy in acute rejection after liver transplantation in patients, liver tissues from were collected from liver transplant recipients with acute rejection (*n* = 10) and control individuals (*n* = 5). The CD8^+^ T cell infiltration and autophagy levels were determined by immunofluorescent staining. The results showed that the number of CD8^+^ T cells that infiltrated liver graft tissues from patients with acute rejection was greater than that found in the liver tissues from patients without acute rejection (Figure 1A), and the CD8^+^ T cell density was positively correlated with the severity of acute rejection (Figure 1C). The autophagy-specific protein LC3 was mainly expressed in the stromal cells and was weakly expressed in parenchymal cells (Figure 1A), and LC3 expression was correlated positively with the severity of acute rejection (Figure 1D). Importantly, immunofluorescent double staining confirmed that most of the LC3-positive cells in liver graft tissues also expressed the T cell marker CD8 (Figure 1B). The autophagy levels in CD8^+^ T cells isolated from peripheral blood were further confirmed by western blotting, as shown in Figure 1E. The LC3II/I protein expression level in the rejection patients was significantly higher than that found in the control individuals and was consistent with the immunohistochemical results.

### Autophagy Was Strongly Induced in CD8^+^ T Cells in a rat Acute Hepatic Rejection Model

**Figure 2:**
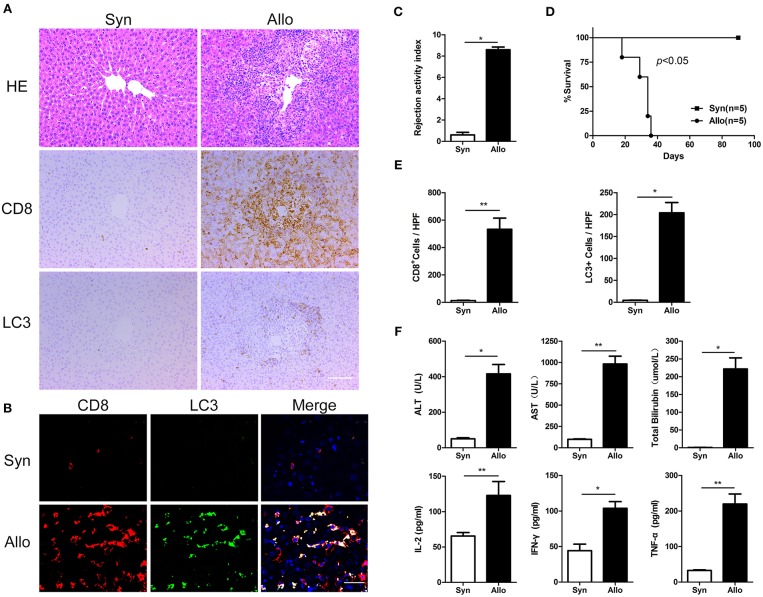
Autophagy was strongly induced in CD8^+^ T cells in a rat acute hepatic rejection model All samples were collected on day 14 after liver transplantation. **(A)** Paraffin-embedded liver grafts were stained with H&E or IHC for CD8 and LC3 in allogenic group and syngeneic group rat recipients. Scale bar, 100 μm. **(B)** Double fluorescent staining for CD8 (red), LC3 (green) and DAPI (blue) in liver grafts. **(C)** Bar graph showing the rejection activity index (RAI) results. **(D)** Survival of rat recipients was compared between allogenic group(*n* = 5) and syngeneic group (*n* = 5) (log-rank, *p* < 0.001). **(E)** Histogram showing the counts of CD8 positive and LC3 positive cells. **(F)** Analysis of serum levels of ALT, AST, and TBIL, and cytokines (IFN-γ, IL-2, and TNF-α). Apart from log-rank used in survival test, student *t*-test was performed for the rest statistical analyses. *p < 0.05, **p < 0.01 compared between groups.

We established an acute rejection model to determine the role of autophagy during rejection and used a syngeneic group as the control. Compared with homogenic recipients, the grafts in allogenic rats showed severe damage, as demonstrated by notable increases in liver injury parameters (serum ALT, AST, and TBIL) and inflammatory cytokines (IL-2, IFN-γ, and TNF-α) (Figure 2F), and these findings were further confirmed by histological assays, which revealed high rejection activity index (RAI) scores and an increased number of graft-infiltrating lymphocytes (Figures 2A,C). The survival time of allogeneic recipients was significantly decreased (Figure 2D). The immunohistochemical staining of serial graft sections showed that the infiltrated lymphocytes were mostly CD8^+^ T cells, and these cells expressed high levels of the autophagy-specific protein LC3 (Figures 2A,E). Moreover, immunofluorescent double staining results showed that the number of LC3-positive in the CD8^+^ T cell population was substantially increased remarkably increased in allogenic allografts (Figure 2B), indicated that CD8^+^ T cells showed increased autophagy during AR.

### Inhibition of Autophagy Significantly Prolonged Recipient Survival

**Figure 3:**
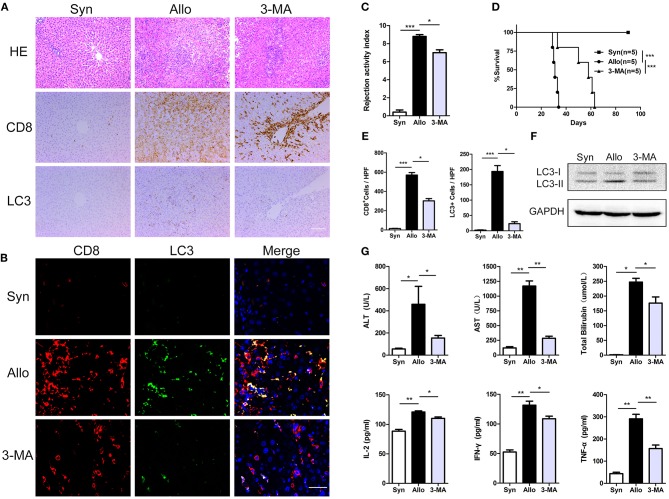
Inhibition of autophagy significantly prolonged recipient survival. All samples were collected on day 14 after liver transplantation. **(A)**, Hematoxylin and eosin staining and immunostaining for CD8 and the autophagy marker LC3 in allogenic group, allogenic group and 3-MA group rat recipients. Scale bar, 100 μm. **(B)** Double fluorescent staining for CD8 (red), LC3 (green) and DAPI (blue) in liver grafts. **(C)** Bar graph showing the rejection activity index (RAI) results in three groups. **(D)** Survival of rat recipients was compared between three groups (log rank, *p* < 0.001). **(E)** Histogram showing the counts of CD8 positive and LC3 positive cells in three groups. **(F)** Expression of LC3 in CD8^+^ T cells isolated from liver grafts was further determined by western blot. **(G)** Analysis of serum levels of ALT, AST, and TBIL, and cytokine (IFN-γ, IL-2, and TNF-α). Apart from log-rank used in survival test, the rest statistical analyses were performed by student *t*-test, *p < 0.05, **p < 0.01, ***p < 0.001 compared between groups.

To determine the effect of CD8^+^ T cell autophagy on acute rejection, allogeneic rat recipients were pre-treated with the autophagy inhibitor 3-MA by intraperitoneal injection. Blood, spleen and liver graft samples were collected on postoperative day (POD) 14. Compared with untreated allograft recipients, 3-MA-treated recipients notably reduced CD8^+^ T cell autophagy, as measured by the number of LC3-positive cells in liver grafts (Figures 3A,B,E), and this finding was further confirmed by western blot (Figure 3F). These changes were accompanied by a reduction in liver rejection histological injury (Figure 3C) and decreased liver enzyme and inflammatory cytokine levels in the blood (Figure 3G). Furthermore, the survival time of the 3-MA-pretreated recipients was significantly longer than that of untreated rats (median survival time of 62 days vs. 33 days) (Figure 3D). These results indicate that the inhibition of autophagy exerts an overall protection in grafts.

### Autophagy Inhibition Accelerated the Apoptosis of CD8+ T Lymphocytes

**Figure 4:**
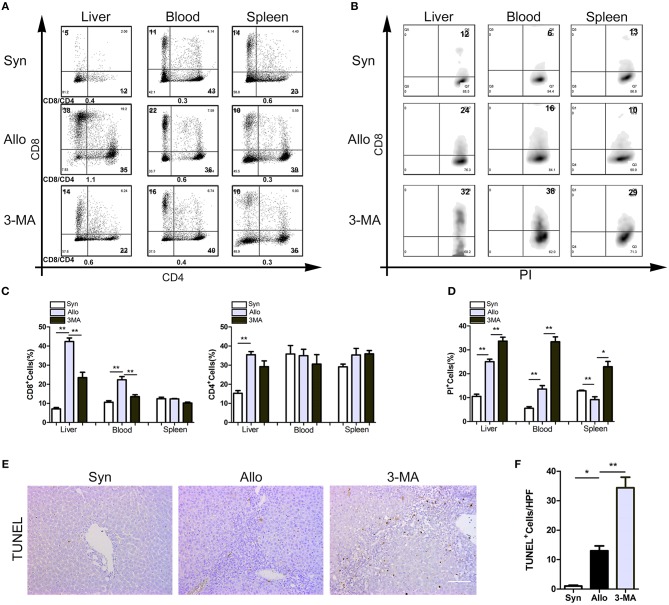
Autophagy inhibition accelerated the apoptosis of CD8^+^ T lymphocytes. All samples were collected on day 14 after liver transplantation. **(A)** Percentage of T cell subset in total graft infiltrating T cells by flow cytometry. **(B)** Flow analyses of percentage of CD8^+^ T cells stained with PI. **(C)** Histogram showing the frequencies of CD8^+^ T cells and CD4^+^ T cells. **(D)** Histogram showing the percentage of apoptotic CD8^+^ T cells. **(E,F)** TUNEL staining showing the apoptotic activity of lymphocytes in liver graft. Scale bar, 100 μm. All statistical analyses were performed by student *t*-test, *p < 0.05, **p < 0.01 compared between groups.

Mononuclear cells isolated from the blood, spleen and liver grafts of rat recipients were obtained on POD14, and a flow cytometry analysis showed that the peripheral blood and allografts of 3-MA-pretreated rats exhibited similar frequencies of CD4^+^ T cells but less frequencies of CD8^+^ T cells compared with those of untreated allograft recipients (Figures 4A,C). We then examined the apoptosis levels of CD8^+^ T cells in the autophagy-inhibited recipients. The results showed that the frequencies of apoptotic CD8^+^ T cells in the blood, spleen and liver were significantly higher in the 3-MA pre-treated group (Figures 4B,D). TUNEL staining further confirmed the apoptotic frequency of lymphocytes from 3-MA-pretreated rats was markedly increased compared with that of lymphocytes from untreated allograft recipients (Figures 4E,F). These results suggest that the inhibition of CD8^+^ T cell autophagy accelerates apoptosis.

### Autophagy Inhibition Impaired the Function and Proliferation of CD8^+^ T Cells

**Figure 5:**
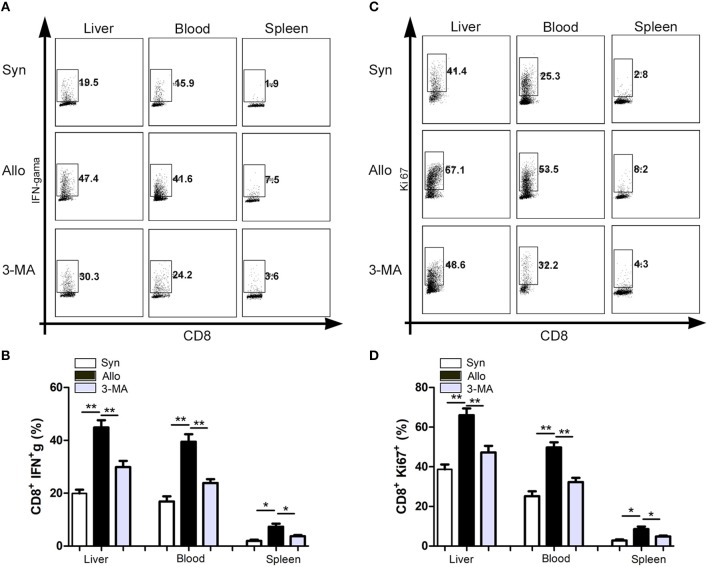
Autophagy inhibition impaired the function and proliferation of CD8^+^ T cells. All samples were collected on day 14 after liver transplantation. **(A,B)** Flow cytometric analysis of IFN-gama secretion by CD8^+^ T cells after restimulation with PMA and Ionomycin. **(C,D)** Flow cytometric analysis of proliferation of CD8^+^ T cells determined by Ki-67 staining. All statistical analyses were performed by student *t*-test, *p < 0.05, **p < 0.01 compared between groups.

We then investigated whether the inhibition of autophagy was correlated with the decreases in the function and proliferation of CD8^+^ T cells. Compared with the untreated allograft recipients, significantly decreased frequencies of IFN-γ-producing CD8^+^ T cells were observed in the blood, graft and spleen o the 3-MA pre-treated recipients (Figures 5A,B). Similarly, the frequencies of Ki67^+^CD8^+^ T cells among the graft-infiltrating lymphocytes were also decreased in the 3-MA pre-treated recipients (Figures 5C,D).

### Enhanced Autophagy Maintained Mitochondria-Mediated CD8^+^ T Cells Survival and Upregulated T Cell Function *in vitro*

**Figure 6:**
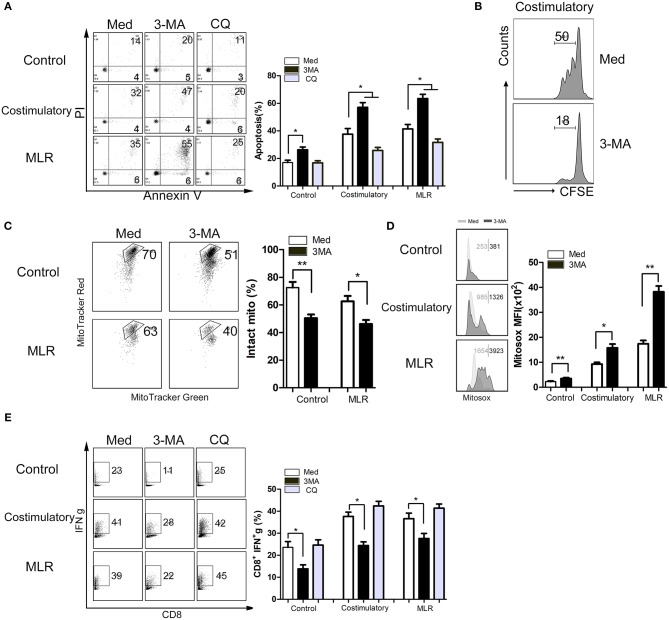
Enhanced autophagy maintains mitochondria-mediated CD8^+^ T cells survival and upregulated function *in vitro*. Lymphocytes isolated from peripheral blood of health donors were cultured in medium alone or activated using anti CD3/28 mAb beads stimulation (costimulatory) or heterologous counterpart (one way MLR) for 12 h. Then lymphocytes were left untreated or treated with 3-MA or CQ for 72 h. **(A)** Levels of apoptotic CD8^+^ T cells with different treatment determined via Annexin V/PI staining. **(B)** CD8^+^ T cells proliferation was determined using CFSE staining. **(C,D)** Intact mitochondria **(C)** and mitochondrial ROS **(D)** were determined by FACS via labeling with MitoTracker Red plus MitoTracker Green, and MitoSOX, respectively. **(E)** IFN-γ production of CD8+ T cells was determined by FACS. All statistical analyses were performed by student *t*-test, *p < 0.05, **p < 0.01 compared between groups.

Our above-described results suggest that enhanced autophagy in CD8^+^ T cells sustains the survival and function of these cells. To further explore the underlying mechanisms, we assessed the survival and functional status of CD8^+^ T cells (cultured alone or activated by anti-CD3/28 mAb beads or heterologous lymphocytes) treated with 3-MA or CQ. Consistent with our results *in vivo*, the inhibition of autophagy by 3-MA significantly decreased the viability and proliferation of CD8^+^ T cells (Figures 6A,B). In contrast, the suppression of autophagic degradation by CQ enhanced cell survival (Figures 6A,B). We then examined whether autophagy status affect mitochondrial function in activated CD^+^8 T cells. The results showed that the inhibition of autophagy in CD8^+^ T cells significantly impaired the stability of mitochondria and increased the production of mitochondrial superoxide anion radicals (Figures 6C,D). Finally, we examined the association between autophagy and pro-inflammatory effects of CD8^+^ T cells. We found that suppression of autophagy also decreased the frequencies of IFN-γ-producing CD8^+^ T cells (Figure 6E). These data indicate that enhanced autophagy of CD8^+^ T cells in turn maintains their survival and function and might result in severe rejection damage to allografts.

## Discussion

It has been well-established that CD8^+^ T cells are crucial effector cells against liver allografts and play a pivotal role in promoting acute rejection after LT (24–26). Consistently, in this study, we also found that CD8^+^ T cells were the main effector cells infiltrating grafts and that their frequency correlated positively with the severity of acute rejection after human and rat liver transplantation. The frequency of alloreactive CD8+ T cells, which is mainly determined by apoptosis and proliferation, is closely associated with acute rejection after LT. In addition to the frequency of alloreactive CD8^+^ T cells, their function is also an important factor that directly affects acute rejection after LT. To date, the mechanisms of apoptosis, proliferation and function of alloreactive CD8^+^ T cells after LT have not been fully elucidated, which largely impedes the development of a definitely effective strategy for inducing immune tolerance. In recent years, numerous studies have reported that autophagy exerts a suppressive effect on apoptosis (27–29) and accelerative effect on proliferation and function of T cells in some contexts (30–32). However, it remains unclear whether autophagy could promote T cell survival in the context of LT and thereby exacerbate acute rejection. With respect to this issue, this study revealed that: (1) CD8^+^ T cell autophagy is strongly induced during acute rejection after LT and is positively correlated with the severity of acute rejection; (2) the autophagy inhibitor 3-MA significantly alleviates the severity of acute rejection after LT and prolongs the survival of liver allografts and recipients; (3) 3-MA can significantly promote CD8^+^ T cell apoptosis, inhibit the function and proliferation of CD8^+^ T cells, and subsequently decreasing the CD8^+^ T cell frequency; and (4) the inhibition of autophagy in activated CD8^+^ T cells largely attenuates the stabilization of mitochondria and increases the production of mitochondrial superoxide anion radicals.

Autophagy, as an intracellular bulk degradation system, is induced mainly under various pathological stresses and has been reported to participate in numerous diseases, including sepsis, neurological disorders, cancer, and ischemia-reperfusion injury (28, 33–35). Some studies have shed light on the role of autophagy in transplantation. Gotoh et al. demonstrated that inhibition of autophagy significantly attenuated the damage of hepatocytes caused by cold ischemia—warm reperfusion injury and improved graft survival in rat LT model (36). Furthermore, Tanemura et al. also found that autophagy inducer rapamycin reduced viability and function of islet β cells and the effects could be abrogated by the use of 3-MA both *in vitro* and *in vivo* (37). However, the role of T cell autophagy in acute rejection after liver transplantation has never been explored. Recent researches have shown that autophagy participates in innate and adaptive immune responses and is involved in some immune disorders (38–40), which indicates that it plays a role in transplant immunity. Thus, we established an acute rejection model in rats to explore the underlying mechanism of autophagy participate in AR. We found that the autophagy inhibitor 3-MA can significantly alleviate the severity of acute rejection after LT and prolong the survival of liver allografts, which suggests that autophagy plays an accelerative role in acute rejection after LT. There is evidence showing that autophagy is substantially induced when T cells proliferate in immune homeostasis (41), indicating that autophagy plays a key role in facilitating T cell survival. Additionally, previous studies have implied that knockout of Atg5 or Vps34, two key autophagy-related factors, significantly inhibited the proliferation of T cells T cells following TCR triggering (14). In view of the fact that CD8^+^ T cells are crucial effector cells against liver allograft during acute rejection, we postulated that 3-MA might alleviate the severity of acute rejection after LT specifically by inhibiting the autophagy-induced maintenance of survival or proliferation of CD8^+^ T cells. As expected, our data showed that CD8^+^ T cell autophagy was strongly induced in patients and rats with acute rejection, and the administration of the autophagy inhibitor 3-MA significantly reduced autophagy and the frequency of infiltrated CD8^+^ T cells in allografts. In general, these results preliminarily suggest that CD8^+^ T cell autophagy contributes to acute rejection after LT by promoting the survival or proliferation of CD8^+^ T cells.

The frequency of alloreactive T cells is positively associated with the severity of acute rejection after LT and is determined by the death and proliferation of alloreactive T cells (42). Particularly, apoptosis is considered the leading mechanism for the death of alloreactive T cells (43), and it is believed that autophagy has the capability of sustaining CD8^+^ T cell survival by inhibiting apoptosis under some conditions. For instance, Oami et al. demonstrated that CD8^+^ T cells were more vulnerable to apoptosis after autophagy blockade in a murine sepsis model (34). Similarly, Pei et al. also reported that autophagy could significantly inhibited the apoptosis pathway of swine fever virus-infected cells by limiting ROS-dependent RIG-I-like receptor (RLR) signaling (44). Therefore, we took advantage of the acute rejection model and a mixed lymphocyte culture assay to further explore whether CD8^+^ T cell autophagy exerted an accelerative effect on acute rejection after LT by inhibiting the apoptosis of CD8^+^ T cells. Consistent with previous studies, our data showed that treatment with autophagy the inhibitor 3-MA substantially promoted the apoptosis of CD8^+^ T cells and decreased their frequency among total lymphocytes. In addition, our experiments both *in vitro* and *in vivo* also suggested that autophagy was able to improve the proliferative capability of CD8^+^ T cells. Furthermore, our *in vitro* experiments also revealed that autophagy plays a key role in sustaining the normal function of activated CD8^+^ T cells. Autophagy is considered to play an important role in sustaining mitochondrial function, which contributes to the proliferation and normal function of CD8^+^ T cells. Consistently, in this study, we found that inhibition of autophagy in activated CD8^+^ T cells largely impaired the stability of mitochondria and increased the production of mitochondrial superoxide anion radicals. Therefore, in the context of liver transplantation, it was also by sustaining mitochondrial function that autophagy promoted the survival, proliferation and function of CD8^+^ T cells. Overall, these results further demonstrated that CD8^+^ T cell autophagy could facilitate acute rejection after LT by inhibiting CD8^+^ T cell apoptosis and promoting the proliferation and function of these cells.

**Figure 7:**
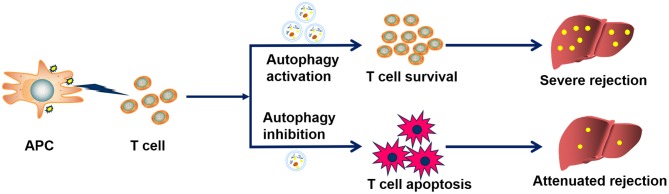
Proposed mechanism by which CD8^+^ T cell autophagy facilitates acute rejection. During acute rejection, APC cells stimulate CD8^+^ T cell activation through the antigen-presenting process and enhanced autophagy promotes the survival and function of activated CD8^+^ T, thereby sustaining the destruction of the graft, eventually leading to the loss of the graft. Thus, measures of inhibiting CD8^+^T cell autophagy may promote CD8^+^ T cell apoptosis and further reduce their cytotoxic effects on the graft, which ultimately contributes to attenuating graft damage and recipient survival.

Based on our data, we proposed a rat model for the role of CD8^+^ T cell autophagy in acute rejection after LT (Figure 7). During acute rejection, APC cells stimulate CD8^+^ T cell activation through the antigen-presenting process and enhanced autophagy promotes the survival and function of activated CD8^+^ T, thereby sustaining the destruction of the graft, eventually leading to the loss of the graft. Thus, measures of inhibiting CD8^+^ T cell autophagy may accelerate CD8^+^ T cell apoptosis and further reduce their cytotoxic effects on the graft, which ultimately contributes to attenuating graft damage and recipient survival.

In conclusion, we first investigated the role of CD8^+^ T cell autophagy in acute rejection after LT and revealed that autophagy might facilitate acute rejection after LT by inhibiting CD8^+^ T cell apoptosis and promoting the proliferation and function of these cells. Thus, therapies targeting CD8^+^ T cell autophagy might be novel strategies for inducing immune tolerance and treating acute rejection after LT.

## Data Availability

The raw data supporting the conclusions of this manuscript will be made available by the authors, without undue reservation, to any qualified researcher.

## Ethics Statement

The study with human samples was approved by the ethics committee of the Third Affiliated Hospital of Sun Yat-sen University. Written informed consents were obtained from all patients and health donors for blood sampling. The experiments with rats were conducted in accordance with the principles and guidelines for the care and use of animals established by Guangdong Pharmaceutical University.

## Author Contributions

GW and XC: conception and design. XC, LW, and QY: data analysis and drafting the manuscript. YY, GC, and GW: manuscript revision. XC, LW, DC, and YD: statistical analysis. GC and GW: funding. XL, GL, JZ, and GW: technical support. XC, QY, GC, and GW: final approval of the submitted version.

### Conflict of Interest Statement

The authors declare that the research was conducted in the absence of any commercial or financial relationships that could be construed as a potential conflict of interest.
